# Impact of Weekly Climatic Variables on Weekly Malaria Incidence throughout Thailand: A Country-Based Six-Year Retrospective Study

**DOI:** 10.1155/2018/8397815

**Published:** 2018-12-04

**Authors:** Manas Kotepui, Kwuntida Uthaisar Kotepui

**Affiliations:** School of Allied Health Sciences, Walailak University, Nakhon Si Thammarat Province, Thailand

## Abstract

*Purpose*. This study aimed to evaluate climatic data, including mean temperature, relative humidity, and rainfall, and their association with malaria incidence throughout Thailand from 2012 to 2017. The correlation of climatic parameters including temperature, relative humidity, and rainfall in each province and the weekly malaria incidence was analyzed using Spearman's rank correlation. The results showed that the mean temperature correlated with malaria incidence (*p* value < 0.05) in 44 provinces in Thailand. These correlations were frequently found in the western and southern parts of Thailand. Relative humidity correlated with malaria incidence (*p* value < 0.05) in 35 provinces. These correlations were frequently found in the northern and northeastern parts of Thailand. Rainfall correlated with malaria incidence (*p* value < 0.05) in 38 provinces. These correlations were frequently found in the northern parts and some western parts of Thailand. The impacts of the mean temperature, relative humidity, and rainfall were observed frequently in specific provinces, including Chiang Mai, Chiang Rai, Trat, Kanchanaburi, Ubonratchathani, and Si Sa Ket. This is the first study to report areas where climatic data are associated with malaria incidence throughout Thailand from 2012 to 2017. These results can map out the climatic change process over time and across the country, which is the foundation for effective early warning systems for malaria, public health awareness campaigns, and the adoption of proper adaption measures that will help in malaria detection, diagnosis, and treatment.

## 1. Introduction

Climate changes are alternations in one or more climate variables, including temperature, relative humidity, rainfall, wind, and sunshine. These changes may impact the survival and reproduction of vectors and the transmission of vector-borne diseases [1]. Malaria, caused by *Plasmodium* spp., is one of the most climate-sensitive mosquito-borne diseases. Climate changes can directly affect malaria transmission by shifting the vector's geographic range and increasing the reproductive and biting rates and by shortening *Plasmodium* spp. incubation period [2]. A previous study successfully developed a system to forecast the probability of malaria incidence using a seasonal timescale multimodel of climate prediction and applied it for the prediction of malaria risk in Botswana [3].

Thailand is a tropical country at the center of the Indochina Peninsula in Southeast Asia, with a total area of approximately 513,000 km^2^, and is located between latitudes 5°37′N to 20°27′N and longitudes 97°22′E to 105°37′E. Thailand is bordered to the north by Myanmar and Laos, to the east by Laos and Cambodia, and to the south by the Gulf of Thailand and Malaysia. Thailand is divided into six regions defined by the National Geographical Committee in 1978, which include northern Thailand's 9 provinces, northeastern Thailand's 20 provinces, western Thailand's 5 provinces, central Thailand's 8 provinces, eastern Thailand's 7 provinces, and southern Thailand's 14 provinces.

The climate of Thailand is under the influence of monsoon winds of a seasonal character, such as the southwest monsoon, which usually starts in mid-May and ends in mid-October, and the northeast monsoon, which normally starts in mid-October and ends in mid-February [4]. Because of the effect of the location and monsoons, Thailand has a relatively high temperature and humidity all year-round, which is ideal for the reproduction of *Anopheles* mosquitoes.

Exploring the relationships between climate change and malaria transmission in Thailand is the first step in developing effective early warning systems for malaria because malaria has been endemic for more than 10 consecutive years in some areas of Thailand. The objective of this study is to clarify the relationship between climate variables and malaria incidence dynamics throughout the 77 provinces of Thailand between 2012 and 2017.

## 2. Materials and Methods

### 2.1. Malaria Incidence in Thailand

Weekly malaria incidences between 2012 and 2017 were retrieved from the Bureau of Vector-Borne Diseases, Ministry of Public Health, Thailand [5], which is available to the public at http://www.thaivbd.org/n/home. Some missing data from the website were requested directly from the Bureau of Vector-Borne Diseases. These data contained the weekly incidence of malaria from all 77 provinces throughout Thailand (52–53 weeks per year).

### 2.2. Climatic Data of Thailand

The daily climatic data were derived from measurements of mean temperature, relative humidity, and rainfall from all weather stations across 77 provinces of Thailand between 2012 and 2017. The climatic data were retrieved from the Thai Meteorological Department. The climatic data included mean temperature (°C), relative humidity (percent), and rainfall (milliliter) in all 77 provinces. The daily climatic data were then transformed using Microsoft Office Excel (Microsoft Corporation, Redmond, VA, USA) to weekly climatic datasets to analyze with weekly malaria incidence data provided by the Bureau of Vector-Borne Diseases.

### 2.3. Correlation of Climatic Parameters and Malaria Incidence in Thailand

The correlation of climatic parameters, including temperature, relative humidity, and rainfall in each province, and the weekly malaria incidence was performed by Spearman's rank correlation. Any province correlated with climatic data was mapped by color in the Thailand map according to the number of years it correlated with malaria incidence. For example, provinces with malaria incidence for more than six years were indicated with a dark color and provinces with malaria incidence for only one year were indicated with a light color.

All statistical analyses were performed using SPSS Statistics for Windows, version 17.0 (SPSS Inc., Chicago, IL, USA). The results are considered statistically significant when *p* values are less than 0.05. The figures were created using Microsoft Office Excel and Microsoft Office PowerPoint (Microsoft Corporation, Redmond, VA, USA).

## 3. Results

### 3.1. Malaria Incidence

**Figure 1:**
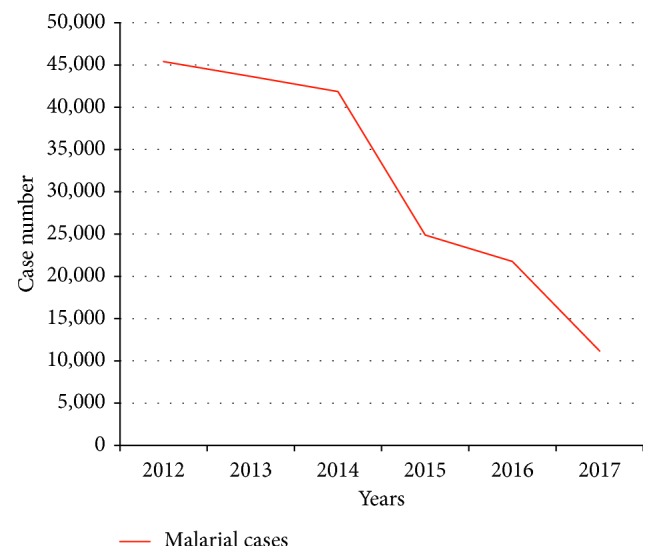
Trend of malaria incidence in Thailand from 2012 to 2017.

According to reports from the Bureau of Vector-Borne Diseases for 2012–2017, the largest malaria outbreak occurred in 2012 with 45,413 cases, and incidence rates continuously decreased year to year to 11,179 cases in 2017.  Figure 1 shows the number of malaria cases in Thailand between 2012 and 2017.

**Figure 2:**
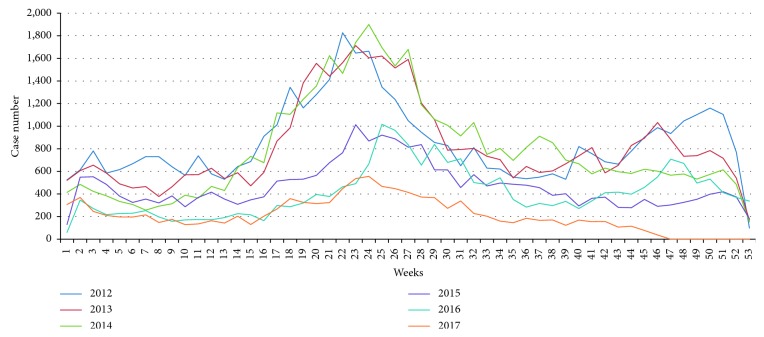
Weekly malaria incidence rates in Thailand from 2012 to 2017.

Malaria incidence rates in each year began to rise in the 15^th^ week of the year and peaked in the 22–28^th^ weeks (Figure 2). It is evident from this figure that malaria incidence and the amount of rainfall are correlated.

### 3.2. Correlation between Mean Temperature and Malaria Incidence in Thailand

**Figure 3:**
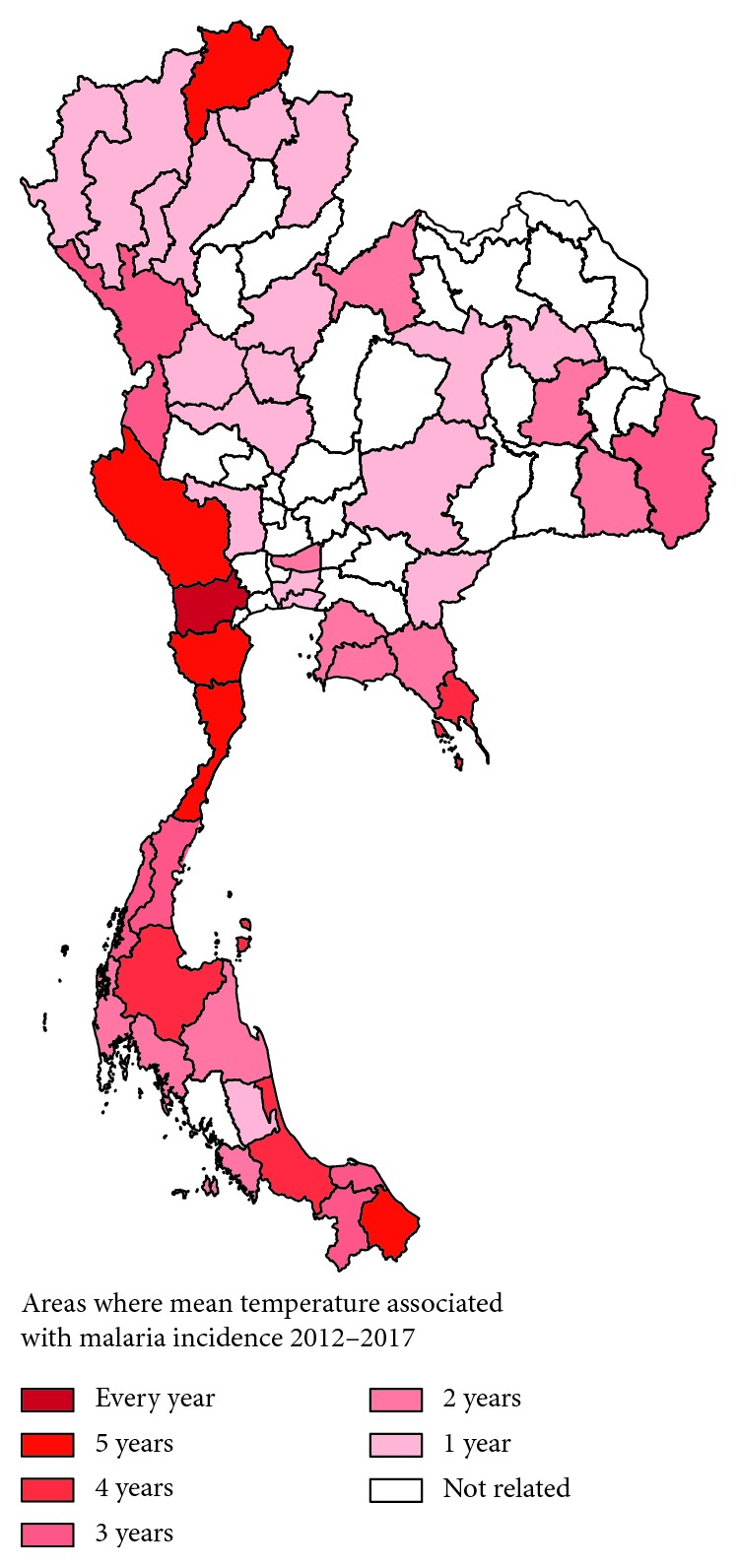
Areas where mean temperature positively correlated with malaria incidence rates.

The results from the correlation test showed that the mean temperature correlated with malaria incidence rates in 44 provinces (*p* value < 0.05). The frequency correlation between the mean temperature and malaria incidence between 2012 and 2017 is shown in Figure 3. Among those 44 provinces, Ratchaburi Province, located in the western part of Thailand, showed a correlation with mean temperatures and malaria incidence rates for six consecutive years between 2012 and 2017 (*p* value < 0.0001). Mean temperatures in Kanchanaburi, Phetchaburi, and Prachuap Khiri Khan in western Thailand and Narathiwat in southern Thailand correlated with malaria incidence rates over five consecutive years between 2012 and 2016 (*p* value < 0.05). Mean temperatures in Chiang Rai, located in the northern part of Thailand, also correlated with malaria incidence rates over five consecutive years between 2013 and 2017 (*p* value < 0.05).

### 3.3. Correlation between Relative Humidity and Malaria Incidence in Thailand

**Figure 4:**
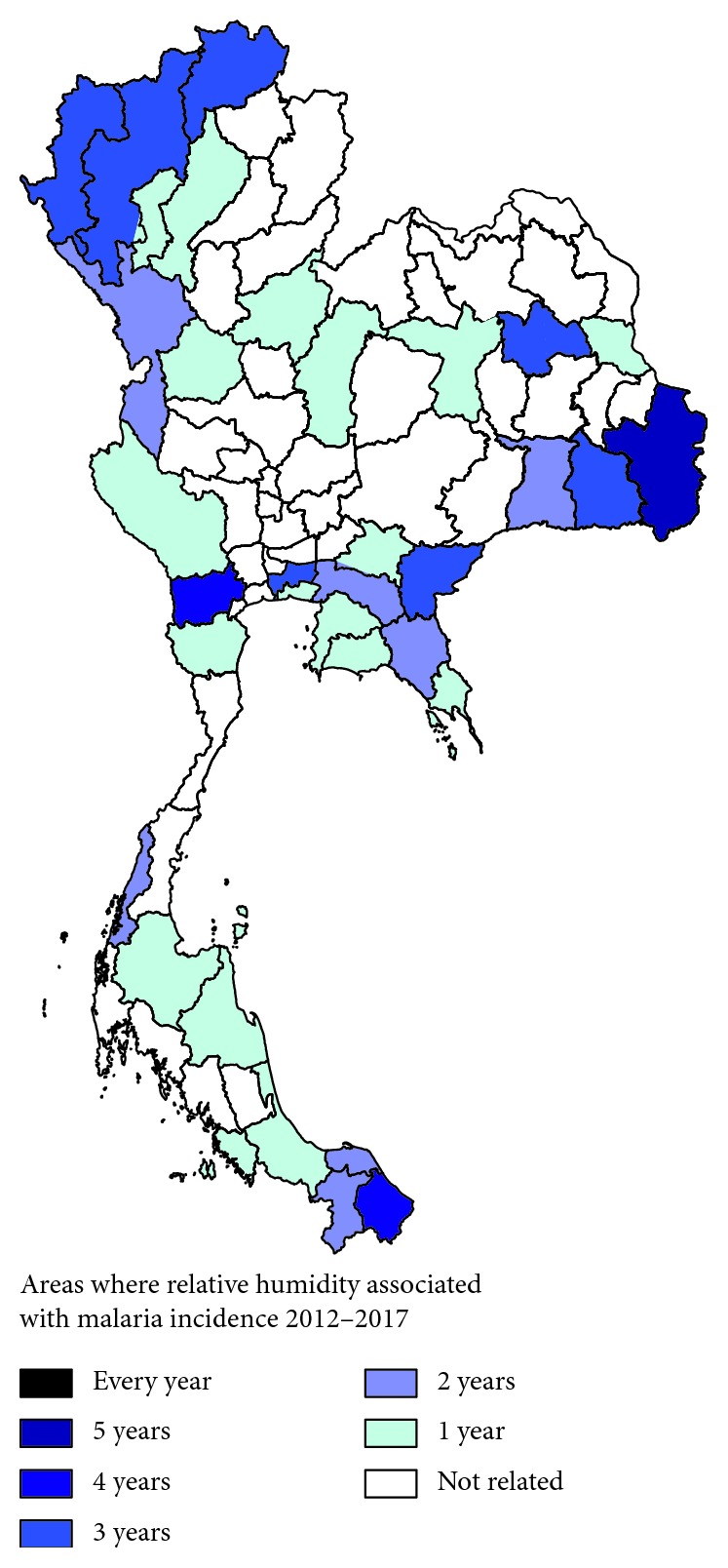
Areas where relative humidity correlated with malaria incidence rates from 2012 to 2017.

The results of the correlation test showed that relative humidity correlated with malaria incidence in 35 provinces (*p* value < 0.05). The frequency correlation between relative humidity and malaria incidence rates in 2012–2017 is shown in Figure 4. Among those provinces, Ubonratchathani, a province located in the northeastern region of Thailand, showed a correlation with relative humidity and malaria incidence over five consecutive years between 2012 and 2017 (*p* value < 0.0001). Relative humidity in Ratchaburi, a province located in western Thailand, correlated with malaria incidence rates over four consecutive years between 2014 and 2017 (*p* value < 0.05). Relative humidity in Narathiwat, a province located in southern Thailand, correlated with malaria incidence rates in 2012, 2013, 2015, and 2016 (*p* value < 0.05).

### 3.4. Correlation between Rainfall and Malaria Incidence in Thailand

**Figure 5:**
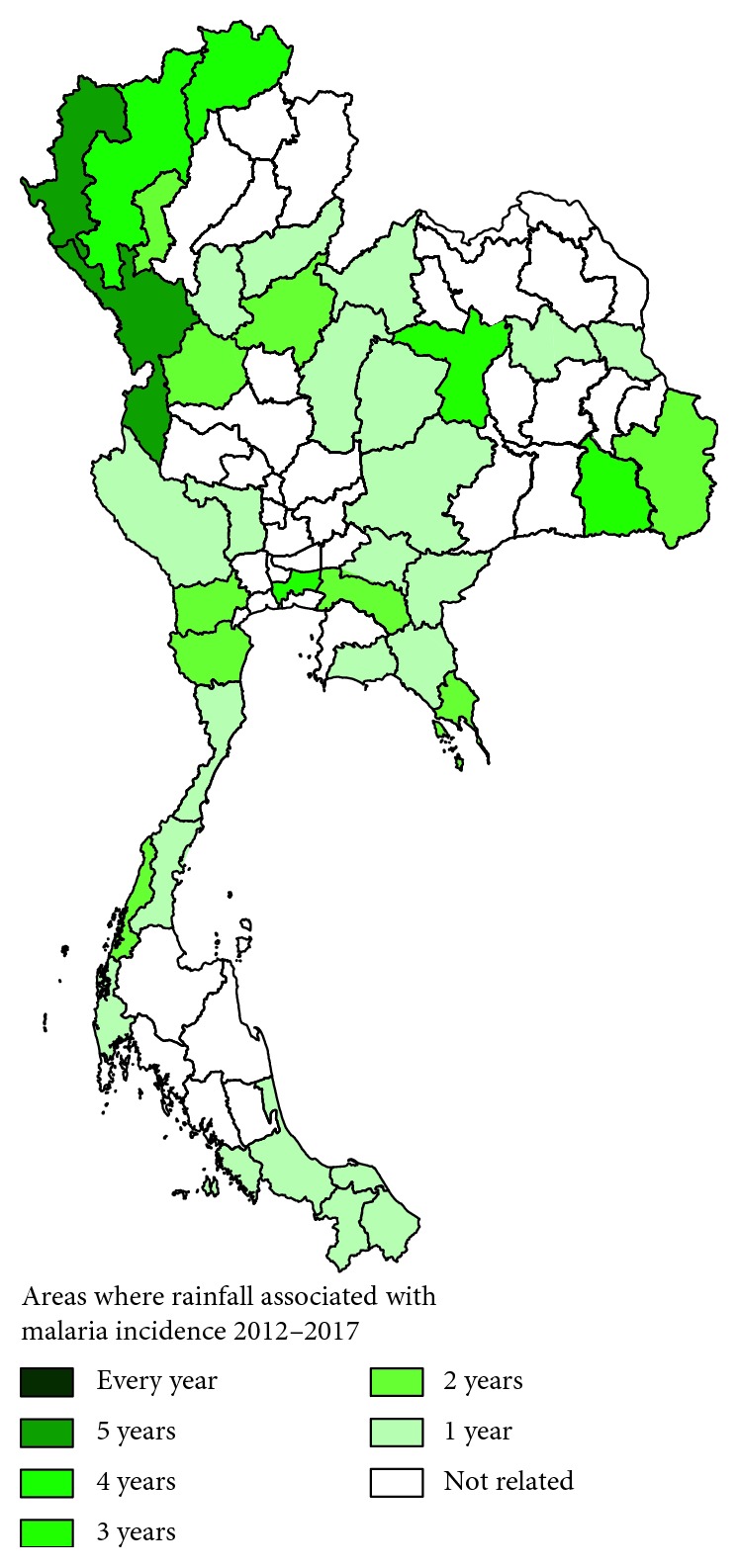
Areas where rainfall correlated with malaria incidence rates from 2012 to 2017.

The results of the correlation test showed that rainfall correlated with malaria incidence in 38 provinces (*p* value < 0.05). The frequency correlation between 2012 and 2017 is shown in Figure 5. Among those provinces, Tak, a province located in western Thailand, showed a correlation with rainfall and malaria incidence rates over five consecutive years between 2013 and 2017 (*p* value < 0.05). Relative humidity in Mae Hong Son, a province located in northern Thailand, correlated with malaria incidence rates over five consecutive years between 2012 and 2016 (*p* value < 0.05). Relative humidity in Chiang Rai, a province located in northern Thailand, correlated with malaria incidence rates over four consecutive years between 2013 and 2016 (*p* value < 0.05). Relative humidity in Chiang Mai, another province in northern Thailand, correlated with malaria incidence rates in 2012, 2015, 2016, and 2017 (*p* value < 0.05).

### 3.5. Dynamic Alteration of Provinces Where Temperature, Relative Humidity, and Rainfall Correlated with Malaria Incidence Rates between 2012 and 2017

**Figure 6:**
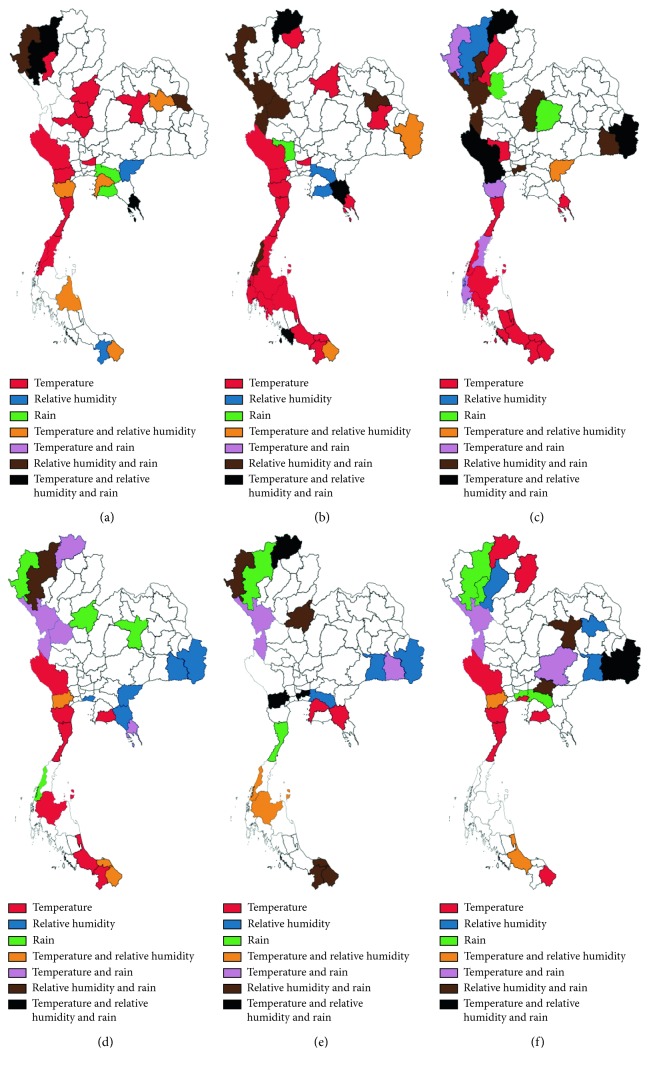
Areas where mean temperature, relative humidity, and rainfall correlated with malaria incidence rates. (a) 2012; (b) 2013; (c) 2014; (d) 2015; (e) 2016; (f) 2017.

Provinces with climatic variables associated with malaria incidence rates from 2012 to 2017 are shown in Figure 6. The mean temperature alone greatly affected malaria incidence rates in western Thailand in the following locations: Kanchanaburi in 2012, 2013, 2015, and 2017; Ratchaburi in 2012, 2013, and 2017; Phetchaburi in 2013, 2015, and 2017; and Prachuap Khiri Khan in 2012, 2013, 2014, 2015, and 2017. It also affected malaria incidence in southern Thailand, such as in Chumphon in 2012 and 2013; Ranong in 2012 and 2014; Surat Thani, Songkhla, and Yala in 2013, 2014, and 2015; Nakhon Si Thammarat and Phang-Nga in 2013; Krabi and Pattani in 2013 and 2014; Phatthalung in 2014; and Narathiwat in 2014 and 2017. It also affected malaria incidence in central Thailand, such as in Nakhon Sawan, Phichit, and Phitsanulok in 2012, Pathum Thani in 2012 and 2013, and Bangkok in 2017. It also affected malaria incidence in eastern Thailand, such as in Trat in 2013 and 2014, Rayoung in 2015 and 2017, and Chanthaburi and Chon Buri in 2016. It also affected malaria incidence in northern Thailand, such as in Lamphun in 2012, Phayao in 2013, Lampang in 2014, and Nan and Chiang Rai in 2017. Lastly, it affected malaria incidence in northeastern Thailand, such as in Khon Kaen in 2012 and Roi Et in 2013.

The relative humidity alone highly affected malaria incidence rates in eastern Thailand in Sa Kaeo in 2012 and 2015, Chachoengsao in 2013 and 2016, Rayong in 2013, and Chanthaburi in 2015. It affected malaria incidence rates in northeastern Thailand in Ubon Ratchathani in 2014 and 2016, Si Sa Ket in 2015, Surin in 2016 and 2017, and Kalasin in 2017, as well as in northern Thailand in Chiang Mai in 2014 and Lampang in 2017.

The rainfall alone greatly affected malaria incidence rates in eastern Thailand, such as in Chachoengsao in 2012 and 2017 and Rayong in 2012. It also affected malaria incidence rates in central Thailand, such as in Suphan Buri in 2013, Sukhothai in 2014, Phitsanulok in 2015, and Bangkok in 2017. Some provinces in northeastern Thailand were also affected by rainfall, such as in Chaiyaphum in 2014 and Khon Kaen in 2015. Some provinces in northern Thailand were also affected by rainfall, such as in Mae Hong Son in 2015, Chiang Mai in 2016 and 2017, and Lamphun in 2017.

In some provinces, two climatic variables were correlated with malaria incidence rates. The mean temperature and relative humidity affected malaria incidence rates in the following locations: northeastern Thailand: in Kalasin in 2012 and Ubon Ratchathani in 2014; eastern Thailand: in Chon Buri in 2012 and Sa Kaeo in 2014; western Thailand: in Phetchaburi in 2012 and Ratchaburi in 2015 and 2017; and southern Thailand: in Narathiwat in 2012, 2013, and 2015; Nakhon Si Thammarat in 2012; Surat Thani and Ranong in 2016; Pattani in 2015; and Songkhla in 2017.

Temperature and rain affected malaria incidence rates in the following locations: northeastern Thailand: in Si Sa Ket in 2016 and Nakhon Ratchasima in 2017; eastern Thailand: in Trat in 2015; western Thailand: in Tak in 2015, 2016, and 2017; Phetchaburi in 2014; and Kamphaeng Phet in 2015; southern Thailand: in Chumphon and Phang-Nga in 2014; and northern Thailand: in Mae Hong Son in 2014 and Chiang Rai in 2015.

Relative humidity and rain affected malaria incidence rates in the following locations: northeastern Thailand: in Mukdahan in 2012, Kalasin in 2013, and Khon Kaen in 2017; eastern Thailand: in Prachin Buri in 2017; western Thailand: in Tak in 2013 and 2014; southern Thailand: in Narathiwat, Pattani, and Yala in 2016; northern Thailand: in Mae Hong Son in 2012, 2013, and 2016; Lamphun in 2014; and Chiang Mai in 2015; and central Thailand: in Kamphaeng Phet in 2013; Phetchabun in 2014; Phitsanulok in 2016; and Bangkok in 2014 and 2016.

Provinces where all climatic parameters including mean temperature, relative humidity, and rainfall affected malaria incidence were found include the following: northern Thailand: in Chiang Mai in 2012 and Chiang Rai in 2013, 2014, and 2016; northeastern Thailand: in Ubon Ratchathani and Si Sa Ket in 2014 and 2017; western Thailand: in Kanchanaburi in 2014 and Ratchaburi in 2016; eastern Thailand: in Trat in 2012 and Chanthaburi in 2013; central Thailand: in Bangkok in 2016; and southern Thailand: in Satun in 2013.

## 4. Discussion

An association between malaria incidence rates and climatic factors has been established. This study examined the impact of climatic variables, including mean temperature, relative humidity, and rainfall, on malaria incidence throughout 77 provinces in six regions of Thailand. The initial findings showed that the mean temperature correlated with malaria incidence rates in 44 provinces in Thailand. These correlations were frequently found in western and southern Thailand. This was because the temperatures in southern Thailand are generally mild throughout the year as a result of being in the coastal area [4], and western Thailand usually has long periods of warm weather because of its inland nature and tropical latitude [4]. These specific locations with suitable temperatures can affect the spatial-temporal distribution of malaria vectors. Temperature is a key driver that determines transmission intensity, including the development and biting rates of *Anopheles* mosquitoes, as well as the development and survival rates of the malarial parasites within the mosquitoes [6, 7]. A previous study also indicated that rising temperatures in low-latitude regions may lead mosquitoes to find new habitats in mid- or high-latitude regions, leading to geographical expansion or shifts in disease transmission [1]. Moreover, a study reported an association between interannual variability in temperature and malaria transmission in the African highlands [8]. Another study in Southeast Iran showed that as the mean, maximum, and minimum monthly temperature increased, the incidence rate of malaria increased significantly [9]. A study in China indicated that mean temperature was also associated with malaria cases over long periods of time [10]. Studies conducted in Tibet and Ethiopia showed strong positive and significant correlations between malaria occurrence and relative humidity, rainfall, and temperature [11, 12].

Both *Anopheles* and *Plasmodium* are sensitive to temperature. Their life stage is dependent on temperature both in development and mortality rates [13]. The previous finding showed that temperatures exceeding 33°C–39°C may limit the development of *P. falciparum* and *P. vivax* [2]. Moreover, a study found that the extrinsic incubation period (EIP) of *P. falciparum* is reduced from 26 days at 20°C to 13 days at 25°C [14]. This means a high development rate of *Plasmodium* and biting rate of *Anopheles* may frequently be found.

Recent research found that *Anopheles* mosquitoes and malarial parasites were influenced not only by the average temperature but also by the temperature fluctuation of the low mean temperatures during the day, which helps to speed up biological processes, whereas fluctuation around the high mean temperatures acts to slow processes down [15]. *P. falciparum* transmission is limited by temperatures below 16°–19°C, whereas *P. vivax* development can occur at temperatures as low as 14.5°–15°C. Parasite development cannot occur with temperatures above 33°–39°C for *P. falciparum* and *P. vivax* [16].

The second result of this study showed that relative humidity correlated with malaria incidence (*p* value < 0.05) in 35 provinces. These correlations were frequently found in the northern and northeastern provinces of Thailand. Humidity was found to affect malarial parasite development in *Anopheles* mosquitoes [17]. Relative humidity affected malaria transmission by impacting the activity and survival of mosquitoes. A study reported that mean monthly relative humidity under 60% causes a shortened lifespan in malaria vector mosquitoes, which results in low malaria transmission rates [18], and a relative humidity of less than 10% is fatal [19]. The results of this study correlated with those of a previous study, showing that relative humidity was positively associated with malaria incidence rates from the same month [20, 21]. A previous study in China showed that relative humidity was associated with *P. vivax* and *P. falciparum* over 8–10 weeks and 5–8 weeks, respectively [10]. Moreover, a study conducted in Yongcheng, China, between 2006 and 2010 demonstrated temperature and relative humidity as the main drivers of malaria transmission [22].

The third result of this study showed that rainfall correlated with malaria incidence (*p* value < 0.05) in 38 provinces. These correlations were frequently found in northern parts and some western parts of Thailand. The upper parts of Thailand usually have rainy weather because of the onset of the southwest monsoons that lead to intensive rainfall from mid-May until early October in this region. The correlation between rainfall and the prevalence of malaria in Thailand has been reported previously; however, the rainfall data were transformed from the geographical data and the prevalence of malaria was transformed from the overall infection rate of malaria, which was not based on real data of malaria cases and real climatic variables from weather stations across Thailand [23]. A previous study reported that monthly rainfall was negatively correlated with the proportion of patients with malaria hyperparasitemia and the proportion of gametocyte carriers among *P. falciparum* cases [24]. The results of this study concurred with a study in Africa that showed that the correlation of persistent malaria transmission was associated with a higher level of rainfall [25]. A previous experimental study showed that rainfall may affect vector populations at the larval and adult stages. Since *Anopheles* mosquitoes breed in small natural pools of clean water, droughts usually result in decreases in vector populations and malaria transmission by limiting the number and quality of vector breeding sites [26]. However, a study in China indicated that rainfall had a decreasing effect on *P. vivax* [10]. This contrary effect may be due to excessive rainfall affecting the mosquito population because strong rains may sweep away their breeding sites [27].

The fourth result of this study showed that more than one climatic variable was associated with malaria incidence. Mean temperature and relative humidity affected malaria incidence rates in some provinces of northeastern, eastern, western, and southern Thailand. However, mean temperature and rain affected malaria incidence rates in the northeastern, eastern, western, southern, and northern regions of Thailand. This means that the alteration of both mean temperature and rainfall has a higher impact on malaria transmission in Thailand than the alteration of both mean temperature and relative humidity. Therefore, northern Thailand, including Mae Hong Son in 2014 and Chiang Rai in 2015, was not affected. The findings also showed that relative humidity and rainfall affected malaria incidence rates in some provinces in all six regions of Thailand, including northeastern, eastern, western, southern, northern, and central Thailand. This means that the alteration of both relative humidity and rainfall has a higher impact on malaria transmission in Thailand than that of mean temperature and relative humidity or mean temperature and rainfall. Because of this, malaria incidence rates among provinces in central Thailand were also affected. The affected provinces of central Thailand included Kamphaeng Phet in 2013, Phetchabun in 2014, Phitsanulok in 2016, and Bangkok in 2014 and 2016. Lastly, the results also showed that alteration of all three climatic parameters, including mean temperature, relative humidity, and rainfall, affected malaria incidence rates in specific provinces in all 6 regions of Thailand. However, the provinces were different from the two climatic parameters. Similar observations of mean temperature and relative humidity affected malaria incidence rates in the Guangzhou area, China, where both temperature and relative humidity were positively associated with malaria incidence [20]. A study in Kenya reported that hospital admissions for malaria have been associated with rainfall and high maximum temperature [28]. However, high maximum temperature and malaria incidence were not analyzed in this study.

The main limitation of the study was the lack of other climatic data, such as wind, length of sunlight, evaporation, and visibility. However, using one or more climatic variables, including mean temperature, relative humidity, and rainfall, could help to map out the climatic change process through time and across the country, which is the foundation for effective early warning systems for malaria, public health awareness campaigns, and the adoption of proper prevention measures that will help with malaria detection, diagnosis, and treatment.

## 5. Conclusion

This was the first study to report the areas where climatic data were associated with malaria incidence throughout Thailand from 2012 to 2017. These results can map out the climatic change process through time and across the country, which is the foundation for effective early warning systems for malaria, public health awareness campaigns, and adoption of proper prevention measures that will help with malaria detection, diagnosis, and treatment.

## Data Availability

The datasets used during the current study are available from the corresponding author upon reasonable request.

## References

[1] Wu X., Lu Y., Zhou S., Chen L., Xu B. (2016). Impact of climate change on human infectious diseases: empirical evidence and human adaptation. *Environment International*.

[2] Patz J. A., Epstein P. R., Burke T. A., Balbus J. M. (1996). Global climate change and emerging infectious diseases. *JAMA: The Journal of the American Medical Association*.

[3] Thomson M. C., Doblas-Reyes F. J., Mason S. J. (2006). Malaria early warnings based on seasonal climate forecasts from multi-model ensembles. *Nature*.

[4] The Climate of Thailand, https://www.tmd.go.th/en/archive/thailand_climate.pdf

[5] Malaria statistics, http://www.thaivbd.org/n/

[6] Mordecai E. A., Paaijmans K. P., Johnson L. R. (2013). Optimal temperature for malaria transmission is dramatically lower than previously predicted. *Ecology Letters*.

[7] Obsomer V., Dufrene M., Defourny P., Coosemans M. (2013). Anopheles species associations in Southeast Asia: indicator species and environmental influences. *Parasites and Vectors*.

[8] Bouma M. J. (2003). Methodological problems and amendments to demonstrate effects of temperature on the epidemiology of malaria. A new perspective on the highland epidemics in Madagascar, 1972–1989. *Transactions of the Royal Society of Tropical Medicine and Hygiene*.

[9] Mohammadkhani M., Khanjani N., Bakhtiari B., Sheikhzadeh K. (2016). The relation between climatic factors and malaria incidence in Kerman, South East of Iran. *Parasite Epidemiology and Control*.

[10] Hundessa S., Williams G., Li S., Guo J., Zhang W., Guo Y. (2017). The weekly associations between climatic factors and Plasmodium vivax and Plasmodium falciparum malaria in China, 2005–2014. *Transactions of the Royal Society of Tropical Medicine and Hygiene*.

[11] Huang F., Zhou S., Zhang S., Wang H., Tang L. (2011). Temporal correlation analysis between malaria and meteorological factors in Motuo County, Tibet. *Malaria Journal*.

[12] Midekisa A., Beyene B., Mihretie A., Bayabil E., Wimberly M. C. (2015). Seasonal associations of climatic drivers and malaria in the highlands of Ethiopia. *Parasites and Vectors*.

[13] Beck-Johnson L. M., Nelson W. A., Paaijmans K. P., Read A. F., Thomas M. B., Bjornstad O. N. (2013). The effect of temperature on Anopheles mosquito population dynamics and the potential for malaria transmission. *PLoS One*.

[14] Bunyavanich S., Landrigan C. P., McMichael A. J., Epstein P. R. (2003). The impact of climate change on child health. *Ambulatory Pediatrics*.

[15] Paaijmans K. P., Blanford S., Bell A. S., Blanford J. I., Read A. F., Thomas M. B. (2010). Influence of climate on malaria transmission depends on daily temperature variation. *Proceedings of the National Academy of Sciences*.

[16] Patz J. A., Olson S. H. (2006). Climate change and health: global to local influences on disease risk. *Annals of Tropical Medicine & Parasitology*.

[17] Climate Change and Infectious Diseases, http://www.who.int/globalchange/publications/climatechangechap6.pdf

[18] Pampana E. (1969). *A Textbook of Malaria Eradication*.

[19] Yamana T. K., Eltahir E. A. (2013). Incorporating the effects of humidity in a mechanistic model of *Anopheles gambiae* mosquito population dynamics in the Sahel region of Africa. *Parasites and Vectors*.

[20] Li T., Yang Z., Wang M. (2013). Temperature, relative humidity and sunshine may be the effective predictors for occurrence of malaria in Guangzhou, southern China, 2006–2012. *Parasites and Vectors*.

[21] Gunda R., Chimbari M. J., Shamu S., Sartorius B., Mukaratirwa S. (2017). Malaria incidence trends and their association with climatic variables in rural Gwanda, Zimbabwe. *Malaria Journal*.

[22] Zhang Y., Liu Q. Y., Luan R. S. (2012). Spatial-temporal analysis of malaria and the effect of environmental factors on its incidence in Yongcheng, China, 2006–2010. *BMC Public Health*.

[23] Wiwanitkit V. (2006). Correlation between rainfall and the prevalence of malaria in Thailand. *Journal of Infection*.

[24] Nacher M., Carrara V. I., Ashley E. (2004). Seasonal variation in hyperparasitaemia and gametocyte carriage in patients with Plasmodium falciparum malaria on the Thai-Burmese border. *Transactions of the Royal Society of Tropical Medicine and Hygiene*.

[25] Pascual M., Cazelles B., Bouma M. J., Chaves L. F., Koelle K. (2008). Shifting patterns: malaria dynamics and rainfall variability in an African highland. *Proceedings of the Royal Society B: Biological Sciences*.

[26] Gage K. L., Burkot T. R., Eisen R. J., Hayes E. B. (2008). Climate and vectorborne diseases. *American Journal of Preventive Medicine*.

[27] Kuhn K., Campbell-Lendrum D., Haines A., Cox J. (2005). *Using Climate to Predict Infectious Disease Epidemics*.

[28] Githeko A. K., Ndegwa W. (2001). Predicting malaria epidemics in the Kenyan highlands using climate data: a tool for decision makers. *Global Change and Human Health*.

